# Serum Proteomics Reveals Alterations in Protease Activity, Axon Guidance, and Visual Phototransduction Pathways in Infants With *In Utero* Exposure to Zika Virus Without Congenital Zika Syndrome

**DOI:** 10.3389/fcimb.2020.577819

**Published:** 2020-11-18

**Authors:** Janaina Macedo-da-Silva, Lívia Rosa-Fernandes, Raquel Hora Barbosa, Claudia B. Angeli, Fabiana Rabe Carvalho, Renata Artimos de Oliveira Vianna, Paulo C. Carvalho, Martin R. Larsen, Claudete Araújo Cardoso, Giuseppe Palmisano

**Affiliations:** ^1^ GlycoProteomics Laboratory, Department of Parasitology, Institute of Biomedical Sciences, University of Sao Paulo, Sao Paulo, Brazil; ^2^ Laboratory for Structural and Computational Proteomics, Carlos Chagas Institute, Fiocruz, Curitiba, Brazil; ^3^ Maternal and Child Department, School of Medicine, Universidade Federal Fluminense, Niteroi, Brazil; ^4^ Multiuser Laboratory for Research in Nephrology and Medical Sciences (LAMAP), School of Medicine, Universidade Federal Fluminense, Niteroi, Brazil; ^5^ Department of Biochemistry and Molecular Biology, University of Southern Denmark, Odense, Denmark

**Keywords:** Zika virus, late abnormalities, serum proteomics, mass spectrometry, biomarker, Congenital Zika Syndrome

## Abstract

In 2015, ZIKV infection attracted international attention during an epidemic in the Americas, when neurological disorders were reported in infants who had their mothers exposed to ZIKV during pregnancy. World Health Organization (WHO) epidemiological data show that 5 to 15% of neonates exposed to ZIKV in the uterus have complications included in abnormalities related to Congenital Zika Syndrome (CZS). The risk of complications after birth is not well documented, however, clinical evidence shows that 6% of infants exposed to ZIKV during pregnancy have complications present at birth, and this rate rises to 14% when medical monitoring is performed in all exposed infants, regardless of birth condition. Thus, the evaluation and monitoring of all exposed infants are of foremost importance as the development of late complications has been increasingly supported by clinical evidence. The identification of changes in protein profile of infants exposed to ZIKV without CZS could provide valuable findings to better understand molecular changes in this cohort. Here, we use a shotgun-proteomics approach to investigate alterations in the serum of infants without CZS symptoms but exposed to intrauterine ZIKV (ZIKV) compared to unexposed controls (CTRL). A complex pattern of differentially expressed proteins was identified, highlighting the dysregulation of proteins involved in axon orientation, visual phototransduction, and global protease activity in children exposed to ZIKV without CZS. These data support the importance of monitoring children exposed to ZIKV during gestation and without early CZS symptoms. Our study is the first to assess molecular evidence of possible late disorders in children victims of the ZIKV outbreak in the Americas. We emphasize the importance of medical monitoring of symptomatic and asymptomatic children, as apparently unexplained late neurological and eye disorders may be due to intrauterine ZIKV exposure.

## Introduction

Zika is a single-stranded RNA virus belonging to the *Flaviviridae* family that was first isolated in 1947 from a rhesus monkey in Kampala, Uganda (Dick et al., 1952). The vectors of these viruses are infected mosquitoes, also responsible for the spread of important diseases such as dengue fever, West Nile fever, and yellow fever (Noorbakhsh et al., 2019). Between 1960 and 1980, Zika virus (ZIKV) was associated with mild diseases in Asian and African populations, however, in 2013-2014, during an outbreak in French Polynesia, the possibility of transplacental transmission from mother to fetus has been described (Kindhauser et al., 2016; Noorbakhsh et al., 2019). In 2015, Zika virus infection attracted international attention during an epidemic in the Americas (Venancio et al., 2019), due to its association with increasing cases of microcephaly, congenital malformation and other neurological disorders in newborns who had their mothers infected during pregnancy (Kindhauser et al., 2016; Mlakar et al., 2016). In 2016, WHO declared the infection a public health emergency of international interest (PHEIC) (Lowe et al., 2018), including 11,546 exposed pregnant women between 2016 and 2017 only in Brazil (Heukelbach et al., 2016). In addition, data published by the Brazilian Ministry of Health indicate 14,558 suspected cases of congenital microcephaly and others central nervous system (CNS) malformations between 2015 and 2017 (Pan American Health Organization/World Health Organization, 2017).

The set of abnormalities resulting from ZIKV intrauterine infection is called congenital Zika syndrome (CZS) and is characterized by severe neurological damage and loss of intracranial volume (Wheeler, 2018). Clinical evidences of CZS are divided into structural components, such as changes in cranial morphology, and functional components that include neurological impairment (Moore et al., 2017; Mohr et al., 2018). The latest published epidemiological bulletin by the Brazilian Ministry of Health reported 19,000 suspected cases of CZS from November 2015 to May 2020, being 3,534 (18.6%) confirmed and 2,784 cases under investigation (Pan American Health Organization/World Health Organization, 2017). CZS has symptoms that are common to other congenital infections, however, has characteristics not previously seen, including severe microcephaly, macular scarring, congenital contractures, and hypertonia (Moore et al., 2017). In 2016, the suggested Zika virus seroprevalence peak in Brazil was 63% (Netto et al., 2017), and following reports indicate that ZIKV continues to circulate in the cycle of human transmission in Brazil and the Americas (Lowe et al., 2018).

The risk of complications after birth is not well established, however, clinical evidence show that 6% of infants exposed to Zika virus during pregnancy have complications at birth, and this rate rises to 14% when medical monitoring is performed on all exposed infants, regardless of birth condition (Rice et al., 2018; Musso et al., 2019; Souza et al., 2019). In addition, subcortical calcifications and an enlarged ventricle have been demonstrated in exposed children, but who were born without clinical evidence of CZS. Another interesting clinical data shows that 60% of these children have seizures during development (Rice et al., 2018; Souza et al., 2019). Reports of other congenital infections, such as cytomegalovirus and toxoplasmosis, indicate that hearing loss and eye damage can occur from 33 to 44 months after birth for symptomatic and asymptomatic infants, respectively (Dahle et al., 2000). Thus, stressing the importance of evaluating and monitoring not only symptomatic infants, but all exposed ones as the development of late complications has been increasingly supported by reports of clinical evidence (Pomar et al., 2018; Rice et al., 2018; Soares et al., 2019; Valdes et al., 2019).

Upon *in utero* zika virus exposure, 12.5% neonates who tested negative for the infection still presented severe, moderate, or mild complications, such as jaundice, hypotonia, hypertonia, hepatomegaly, and elevated liver enzymes (Pomar et al., 2018). Moreover, zika virus exposure without evidence of microcephaly has also been connected to events of asphyxia, hypersalivation, and reflux. In addition, exposed children had lower weight, length, and fat-free masses in the first three months of life compared to unexposed children (Soares et al., 2019). Zika virus-exposed infants without CZS also showed lower receptive language scores in the first year of life (Valdes et al., 2019), besides multiple cerebral and visual abnormalities, which would not have been identified without prolonged medical monitoring (Rice et al., 2018).

In view of the previously reported clinical data, the identification of molecular changes in infants exposed to ZIKV, but who did not have CZS, is important to understand molecular alterations underlying the occurrence of late abnormalities. Here, we use a shotgun-proteomic approach to investigate molecular markers in the serum of infants exposed to intrauterine ZIKV (ZIKV), but without symptoms of CZS, compared to those not exposed to ZIKV (CTRL). A complex pattern of differentially abundant proteins has been identified, highlighting the deregulation of proteins involved in axon guidance, visual phototransduction and global protease activity in children exposed to ZIKV without CZS. These data support the importance of monitoring children exposed to ZIKV during pregnancy and without early symptoms of CZS.

## Materials and Methods

### Patient Cohort

This study includes 20 infants aged between 3 and 23 months referred to the Pediatrics Service of the Antonio Pedro University Hospital, Universidade Federal Fluminense, Brazil. The cohort was divided in CTRL group (non-exposed to ZIKV and negative maternal qPCR, n = 10) and ZIKV group (positive maternal qPCR, n = 10), which consisted of patients with maternal ZIKV exposure during pregnancy and no clinical evidence of CZS. In both CTRL and ZIKV conditions, the mothers tested negative to other infectious agents (syphilis, toxoplasmosis, rubella, cytomegalovirus, and HIV). This study was approved by the institutional review board and the ethics committee of the Universidade Federal Fluminense (protocol CAAE number 79890517.6.0000.5243) and followed the guidelines of the Declaration of Helsinki. All samples were collected upon informed and written consent from the parents/legal guardians of each participant. All participants were clinically evaluated by a multidisciplinary team and are included in a currently ongoing clinical follow-up program (Vianna et al., 2019). The clinical diagnosis was performed based on the guidelines of the Ministry of Health (Brazil, Ministry of Health, 2017).

### Sample Collection

Venous blood samples were collected in Vacutainer blood collection tubes with clot activator (Becton Dickson, USA) and centrifuged (1,210*g* for 15 min) to obtain the serum. Subsequently, the samples were aliquoted in sterile tubes and frozen at -80°C until further analysis. Clinical data were obtained during the outpatient clinic visit and from the patients’ medical records. Epidemiological and demographic data were retrieved from the questionnaire for investigating suspected cases of microcephaly related to ZIKV infection, made available by the Brazilian Ministry of Health, and applied to all patients.

### Sample Preparation for Mass-Spectrometry Based-Proteomics

Serum samples were depleted using the Multiple Affinity Removal System Spin Depletion Cartridge (Agilent Technologies) as per the manufacturer’s instructions. This approach reduces the levels of the 14 most abundant serum proteins (albumin, IgG, antitrypsin, IgA, transferrin, haptoglobin, fibrinogen, alpha2-macroglobulin, alpha1-acid glycoprotein, IgM, apolipoprotein AI, apolipoprotein AII, complement C3, and transthyretin) by approximately 94%. In parallel, non-depleted serum samples were diluted 10× without any pretreatment. Depleted and non-depleted samples were quantified using the Qubit Protein Assay Kit platform (Invitrogen) according to the manufacturer’s instructions. A total of 20 μg proteins were reduced with 10 mM Dithiothreitol (DTT) at 30°C for 45 min and alkylated with 40 mM of iodoacetamide (IAA) for 30 min at room temperature in the dark. The samples were digested with 10% (m/m) trypsin (Promega) during 16 h at 30°C. Following digestion, all reactions were acidified with 1% (v/v*)* trifluoroacetic acid and tryptic peptides were desalted using C18 in-house stage-tips (3M Empore), dried and suspended in 0.1% formic acid (FA) prior to LC-MS/MS analysis.

### Mass Spectrometry Analysis

For analysis of the depleted samples, an UltiMate 3000 Nanoflow LC system (Thermo Scientific) coupled online to a hybrid Quadrupole-Orbitrap mass spectrometer HF-X (Thermo Fisher Scientific) was used. The peptide mixture was loaded on an in-house packed reversed-phase pre-column (4 cm × 100 μm inner diameter, ReproSil-Pur C18-AQ 5 μm particles) and subsequently eluted onto a 20-cm 75-μm inner diameter analytical column containing ReproSil-Pur C18-AQ 3-μm particles. We applied a 66-min gradient using 0 to 35% solvent B in 40 min, 35 to 45% in 15 min, 45 to 99% B in 3 min and 5% B for 2 min (A = 0.1% FA; B = 90% ACN, 0.1% FA). After each run, the trap column and column were equilibrated with mobile phase A. The Quadrupole-Orbitrap HF-X instrument was set to data-dependent acquisition (DDA) and was operated in a positive mode. Survey scans (350–1,600 m/z) were acquired in the Orbitrap system with a resolution of 120,000 at m/z 200. The 20 most intense ions were sequentially isolated and HCD (Higher Energy Collision Dissociation) fragmented using normalized collision energy. The general mass spectrometric conditions were as follows: 2 kV spray voltage, no sheath and auxiliary gas flow, heated capillary temperature of 275°C, predictive automatic gain control (AGC) enabled, and an S-lens RF level of 40%.

The non-depleted samples were analyzed on an LTQ-Orbitrap Velos ETD (Thermo Fisher Scientific) coupled with Easy NanoLC II (Thermo Scientific). The peptide mixture was loaded on a ReproSil-Pur C18-AQ C18 reversed phase column (4 cm × 100 μm inner diameter, 5 μm particles) and subsequently eluted onto a 20 cm 75 inner diameter analytical column containing ReproSil-Pur C18-AQ 3 μm particles. We applied a 130-min gradient using the from 3 to 28% solvent B in 100 min, 28 to 45% in 20 min, 45 - 95% B in 2 min and 8 min at 95% B (A = 0.1% FA; B = 90% ACN, 0.1% FA). After each run, the trap column and column were equilibrated with mobile phase A. The LTQ-Orbitrap Velos instrument was set to data-dependent acquisition (DDA) and was operated in a positive mode. Survey scans (350–1,500 m/z) were acquired in the Orbitrap system with a resolution of 60,000 at *m/z* 110. The 20 most intense ions, excluding unassigned and 1+ charge state, were sequentially isolated and CID (Collision-induced dissociation) fragmented using normalized collision energy of 35. The general mass spectrometric conditions were as follows: 1.9 kV spray voltage, no sheath and auxiliary gas flow, heated capillary temperature of 280°C, predictive automatic gain control (AGC) enabled, and an S-lens RF level of 65.88%.

### Database Search and Statistical Analysis

The raw files corresponding to the depleted and non-depleted serum were searched using Proteome Discoverer v2.3.0.523 and PatternLab for proteomics v4.1.1.17 (Carvalho et al., 2016) (http://patternlabforproteomics.org/), using the SEQUEST search engine. The *H. sapiens* Swiss-Prot database was downloaded on January 24, 2020; a reversed version of each sequence plus those from 127 common mass spectrometry contaminants was included. Trypsin was used as a cleavage enzyme (fully tryptic and semi-tryptic), allowing a maximum of 2 missed cleavage sites. Cysteine carbamidomethylation and methionine oxidation were considered as a static and dynamic modifications, respectively. In the Proteome Discoverer and PatternLab for Proteomics tools, false Discovery Rate (FDR) was 1% for peptide and protein identification. Label free quantification (LFQ) was performed by applying the extracted ion chromatogram (XIC) area. In PatternLab, the quantitation was performed according to Normalized Ion Abundance Factors (NIAF) as a relative quantitation strategy. NIAF is the equivalent to NSAF (Zybailov et al., 2006), but applied to XIC (Neilson et al., 2011). Statistical analyzes were performed using the Perseus 1.5.3.2 software (Cox and Mann, 2008), Proteome Discoverer, and PatternLab for Proteomics (Carvalho et al., 2016). Differentially regulated proteins and semi-tryptic peptides were determined by applying a t-test with an adjusted p-value <0.1 (Benjamini-Hochberg method).

### Bioinformatics Analysis

For gene ontology (GO) analysis, the tool g: Profiler (Raudvere et al., 2019) was employed. The analyses were performed separately for proteins identified upregulated and downregulated between the groups evaluated. A q-value threshold of 0.05 was used, corrected by the Benjamini-Hochberg method. The Gene Enrichment Analysis (GSEA) (Subramanian et al., 2007) was applied to evaluate enriched pathways using the Reactome platform as reference data. Other parameters were used with configurations: permutation method: genes, minimum number of members: 3, maximum number of members: 84, metric for ranking genes: T-test. The Database for Annotation, Visualization and Integrated Discovery (DAVID) v6.8 (Huang et al., 2009) was used to complement bioinformatics analysis. IceLogo tool (Colaert et al., 2009), BRENDA enzyme database (Placzek et al., 2017), Proteasix (http://www.proteasix.org/) and MEROPS - the Peptidase Database (Rawlings et al., 2018) were used to access and analyze peptidases activity.

### Analysis of Enzyme Activity

To analyze the enzymatic activity of the samples, a gelatin zymography was performed as previously described (Toth and Fridman, 2001). Proteins were resolved electrophoretically in 12% SDS-PAGE containing 0.1% gelatin. To remove the SDS, the gel was incubated two times of 30 min with wash buffer (2.5% Triton X-100, 50 mM Tris HCl, 5 mM CaCl2, 1 μM ZnCl2, and H2O); followed by a wash in water and incubation for 12 h in incubation buffer (Triton x 100 at 1%, Tris HCl 50 mM pH 7.5, 5 mM CaCl2, 1 µM ZnCl2) at 37°C in a water bath. The gels were stained with 0.5% Coomassie blue and MMPs activity was determined by the intensity of the band using the ImageLab 3.0 software. Statistical significance was assessed by Student t-test using Graphpad Prism 5 software.

## Results

### The Serum Proteomic Profile of ZIKV Exposed Children Without CZS Is Altered Compared to Controls

**Figure 1:**
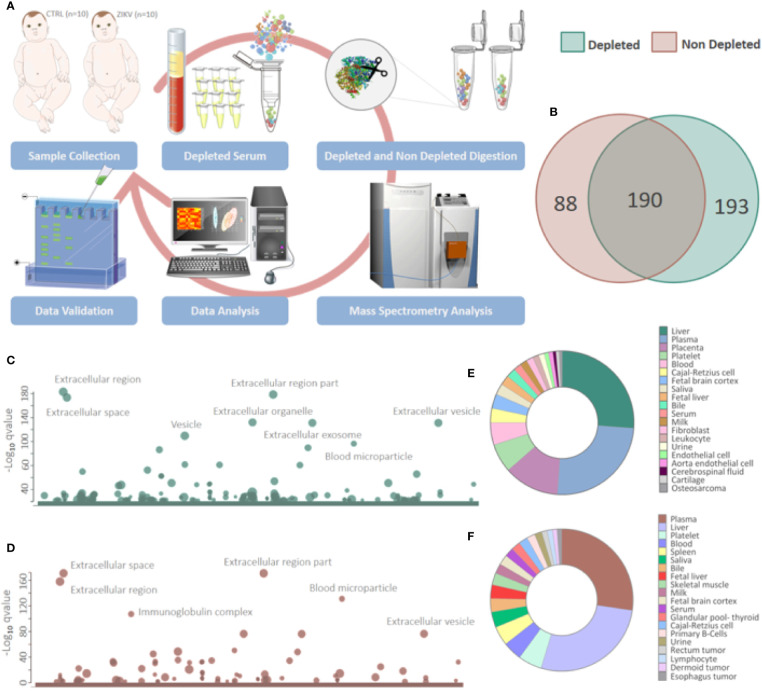
Proteins identified in depleted and non-depleted serum. **(A)** Workflow adopted for sample preparation and data analysis. **(B)** Venn diagram of proteins identified in the depleted and non-depleted serum. Only proteins identified by the Proteome Discoverer and PatternLab for Proteomics software were considered for posterior analysis. **(C, D)** Cellular components attributed to the total proteins identified in depleted and non-depleted treatment were determined using the tool g: Profiler. (q-value < 0.05). **(E, F)** Tissues that express identified proteins were evaluated by the Database for Annotation, Visualization and Integrated Discovery (DAVID) v6.8 platform. Only identifications with a p-value equal to or less than 0.05 and that present at least five proteins were considered.

The serum of infants without symptoms of CZS, but with intrauterine exposure to ZIKV and unexposed controls was evaluated by a proteomic approach based on mass spectrometry, with and without depletion of the 14 most abundant serum proteins **(** **Figure 1A** **)**. The gestational and maternal age was similar between ZIKV and CTRL infants **(** **Supplementary Figures 1A–C** and **Supplementary File 1** **)**. The age of infants varied from 3 to 23 months (CTRL = 14.4 and ZIKV = 13.2) without statistical significance (p-value = 0.3379) **(** **Supplementary Figure 1B** and **Supplementary File 1** **)**. The average head circumference was smaller in the ZIKV group, however, this difference was not statistically significant (p-value = 0.2792) **(** **Supplementary Figure 1D** and **Supplementary File 1** **)**.

All mothers presented rash during the second or third trimester of pregnancy. Boys and girls distribution between CTRL and ZIKV groups are six boys and four girls each, and 75% of 20 families were favela residents. One child of the ZIKV exposed group presented developmental delays and was diagnosed with apraxia of speech and attention deficit hyperactivity disorder. This child had abnormal magnetic resonance findings, with the high signal at the periventricular area in T2 and FLAIR, prominence of perivascular spaces with unspecific aspect, and corpus callosum thinning **(** **Supplementary File 1** **)**.

**Figure 2:**
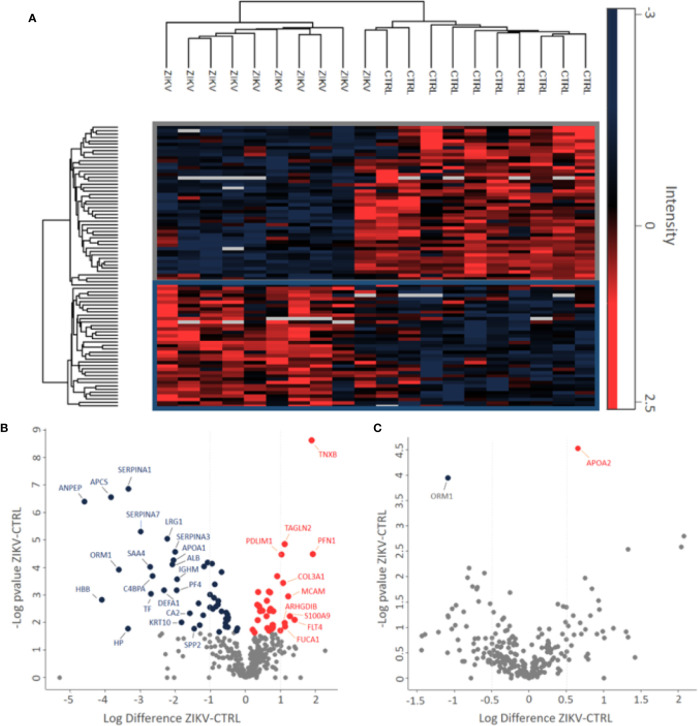
Differently abundant proteins identified in the groups of patients CTRL and ZIKV. **(A)** Heatmap of the proteins regulated in the depleted serum between the evaluated groups. The intensities were normalized using the z-score function. The red and blue colors show upregulated and downregulated proteins, respectively. The blank color indicates that the protein did not show any intensity in the sample. **(B, C)** Volcano plot of depleted and non-depleted serum proteins, respectively. The fold change is represented by the ratio between ZIKV and CTRL protein intensities; negative values indicate greater abundance in the CTRL group compared to the ZIKV group. In **(B)** proteins with fold change > 1 were marked, in **(C)** both regulated proteins were marked.

Proteomics identified a total of 383 and 449 proteins in the depleted serum using the Proteome Discoverer and PatternLab software, respectively (**Supplementary 2**). The 383 common proteins between the two software were selected for further statistical analysis. The identifications in the non-depleted serum were 278 and 438 proteins by Proteome Discoverer and PatternLab for Proteomics, respectively. The 278 proteins identified by both search engines were considered for further analysis. A total of 190 proteins were identified in common between the depleted and non-depleted serum: and 193 and 88 exclusive proteins in the non-depleted and depleted treatment, respectively **(** **Figure 1B** **)**. The Principal Component Analysis (PCA) of depleted **(** **Supplementary Figure 1E** **)** and non-depleted **(** **Supplementary Figure 1F** **)** serum are different, with separation between two distinct groups only in the depleted serum **(** **Supplementary File 1** **)**. GO analysis of each dataset was performed to evaluate the effect of serum depletion on the cellular components (CC) of identified proteins. As expected, the variety of CC identified in the depleted **(** **Figure 1C** **)** and non-depleted **(** **Figure 1D** **)** serum revealed minor variation; however, proteins of the immunoglobulin complex were enriched only in the non-depleted serum. We also evaluated tissues that express the identified proteins **(** **Figures 1E, F** **)**. The depleted serum shows greater diversity in the tissue distribution of identified proteins (**Figure 1E**), with several proteins being expressed in the placenta **(** **Supplementary File 2** **)**. In the depleted serum, 84 proteins were regulated, with 36 upregulated and 48 downregulated in the serum of infants with ZIKV intrauterine exposure compared to controls (**Figure 2A**). A total of 37 proteins showed fold change greater than 1 time (**Figure 2B** and **Table 1** **)** while the non-depleted serum indicated only two regulated proteins, one upregulated and one downregulated (**Figure 2C**, **Supplementary File 3**).

**Table 1:** Differently abundant proteins identified in CTRL and ZIKV patient groups.

Gene name	ZIKV/CTRL Log2 Ratio	Description
PFN1	1.91954	Profilin-1
TNBX	1.88759	Tenascin-X
FLT4	1.40468	Vascular endothelial growth factor receptor 3
S100A9	1.2597	Protein S100-A9
MCAM	1.21672	Cell surface glycoprotein MUC18
FUCA1	1.12497	Tissue alpha-L-fucosidase
TAGLN2	1.11761	Transgelin-2
ARHGDIB	1.11735	Rho GDP-dissociation inhibitor 2
COL3A1	1.07535	Collagen alpha-1(III) chain
PDLIM1	1.03179	PDZ and LIM domain protein 1
B4GALT1	−1.00354	Beta-1,4-galactosyltransferase 1
LAMP1	−1.00966	Lysosome-associated membrane glycoprotein 1
APOM	−1.07515	Apolipoprotein M
FCGR3A	−1.18018	Low affinity immunoglobulin gamma Fc region receptor III-A
GGH	−1.20471	Gamma-glutamyl hydrolase
A2M	−1.3034	Alpha-2-macroglobulin
PEPD	−1.3376	Xaa-Pro dipeptidase
SPP2	−1.46451	Secreted phosphoprotein 24
CA2	−1.58602	Carbonic anhydrase 2
KRT10	−1.82313	Keratin, type I cytoskeletal 10
IGHM	−1.94719	Immunoglobulin heavy constant mu
PF4	−1.95174	Platelet factor 4
SERPINA3	−2.00752	Alpha-1-antichymotrypsin
APOA1	−2.05201	Apolipoprotein A-I
ALB	−2.08527	Serum albumin
LRG1	−2.23184	Leucine-rich alpha-2-glycoprotein
DEFA1	−2.31688	Neutrophil defensin 1
C4BPA	−2.64302	C4b-binding protein alpha chain
TF	−2.69371	Serotransferrin
SAA4	−2.71299	Serum amyloid A-4 protein
SERPINA7	−2.98174	Thyroxine-binding globulin
SERPINA1	−3.34126	Alpha-1-antitrypsin
HP	−3.34659	Haptoglobin
ORM1	−3.60256	Alpha-1-acid glycoprotein 1
APCS	−3.83006	Serum amyloid P-component
HBB	−4.09269	Hemoglobin subunit beta
ANPEP	−4.59327	Aminopeptidase N

Protein ID: Swiss-Prot protein identifier.

Fold change: ratio between ZIKV and CTRL protein intensities; negative values indicate greater abundance in the CTRL group compared to the ZIKV group. Protein description: according to the Swiss-Prot database. All proteins satisfy a q-value < 0.1.

### Disease-Related and Enriched Pathways Analysis

**Figure 3:**
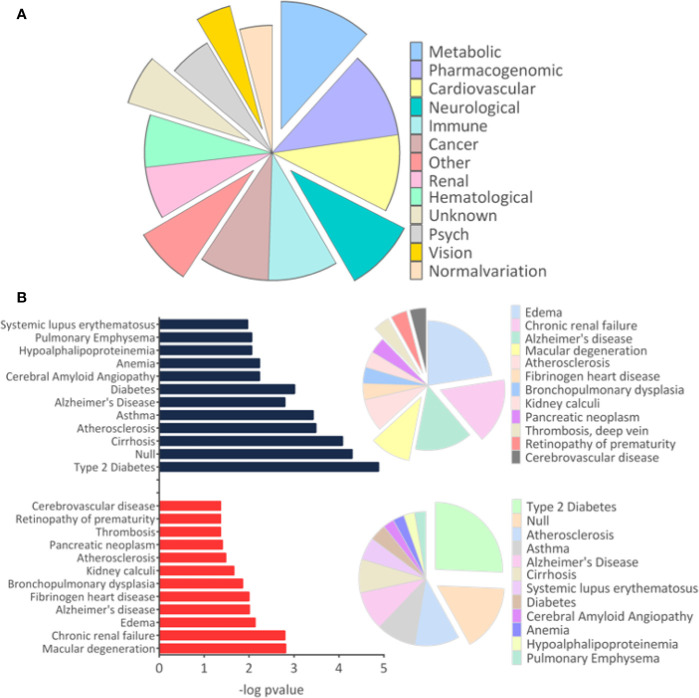
Disease-related proteins. **(A)**. The “Diseases” module in the Database for Annotation, Visualization and Integrated Discovery (DAVID) tool was used to determine the disease classes related to all regulated proteins. **(B)** The analysis for proteins related to diseases was performed separately for upregulated and downregulated proteins. Only tissues and diseases that presented p-value <0.05 were represented in the graph.

The results of disease-related and enriched protein analysis are broad, due to proteins that are shared between multiple pathological processes (**Supplementary File 4**). Indeed, evaluation of diseases related to all differentially regulated proteins showed different classes of disorders, which include metabolic, renal, brain, cardiovascular abnormalities, among others (**Figure 3A**). When evaluation was performed for upregulated proteins, data indicate that proteins associated to macular degeneration, thrombosis, retinopathy of prematurity, and cerebrovascular disease (**Figure 3B**). These data provide insight of important molecular changes that might be related to exposure to ZIKV *in utero*.

**Figure 4:**
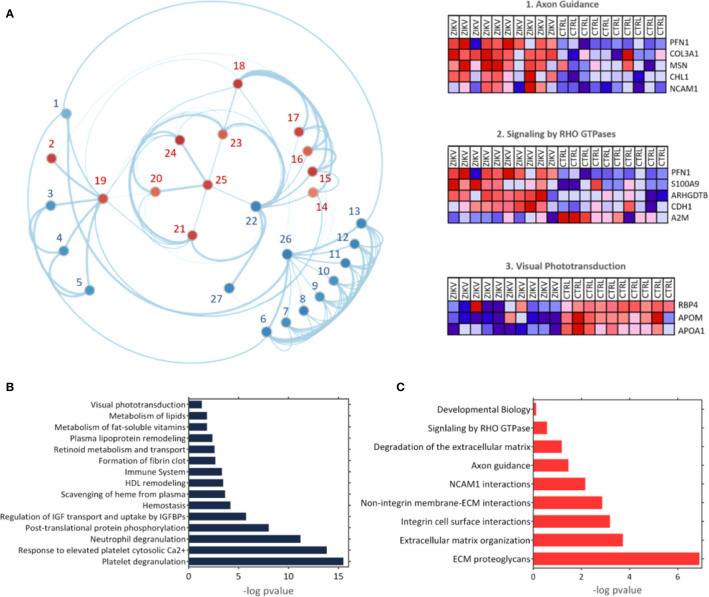
The Gene Enrichment Analysis (GSEA). **(A)** Results obtained with the GSEA analysis; the reference data used were the Reactome platform pathways. The red and blue dots indicate upregulated and downregulated pathways, respectively. The numbered pathways correspond to: 1: Formation of fibrin clot, 2: Signaling by RHO GTPase, 3: Metabolism of fat-soluble vitamins, 4: Retinoid metabolism and transport, 5: Visual phototransduction, 6: Response to elevated platelet cytosolic Ca^2+^, 7: Metabolism of lipids, 8: Scavenging of heme from plasma, 9: Plasma lipoprotein remodeling, 10: HDL remodeling, 11: Post-translational protein phosphorylation,12: Regulation of Insulin-like Growth Factor (IGF) transport and uptake by Insulin-like Growth Factor Binding Proteins (IGFBPs), 13: Platelet degranulation, 14: Degradation of the extracellular matrix, 15: Integrin cell surface interactions, 16: Non-integrin membrane-ECM interactions, 17: ECM proteoglycans, 18: Extracellular matrix organization, 19: Signal Transduction, 20: Developmental Biology, 21: Signaling by Interleukins, 22: Immune System, 23: Cytokine signaling in immune system,24: Axon guidance, 25: NCAM1 interactions, 26: Hemostasis, 27: Neutrophil degranulation. Heatmaps represent proteins related to three chosen pathways: Axon guidance, RHO GTPase signaling, and visual phototransduction. **(B, C)** Upregulated and downregulated pathways, respectively.

The GSEA Reactome analysis showed 27 differently regulated pathways **(** **Figure 4A** **)**. The colors red and blue represent upregulated and downregulated pathways, respectively; heatmaps indicate proteins and their related pathways. Axon guidance and RHO GTPase signaling were identified upregulated in the ZIKV group, while visual phototransduction and retinoid metabolism are downregulated (**Figure 4A**). The pathways were represented in relation to p-value; in positive regulation **(** **Figure 4B** **)** and negative regulation **(** **Figure 4C** **)**. Complete information on the GSEA analysis is available in **Supplementary file 5**.

**Figure 5:**
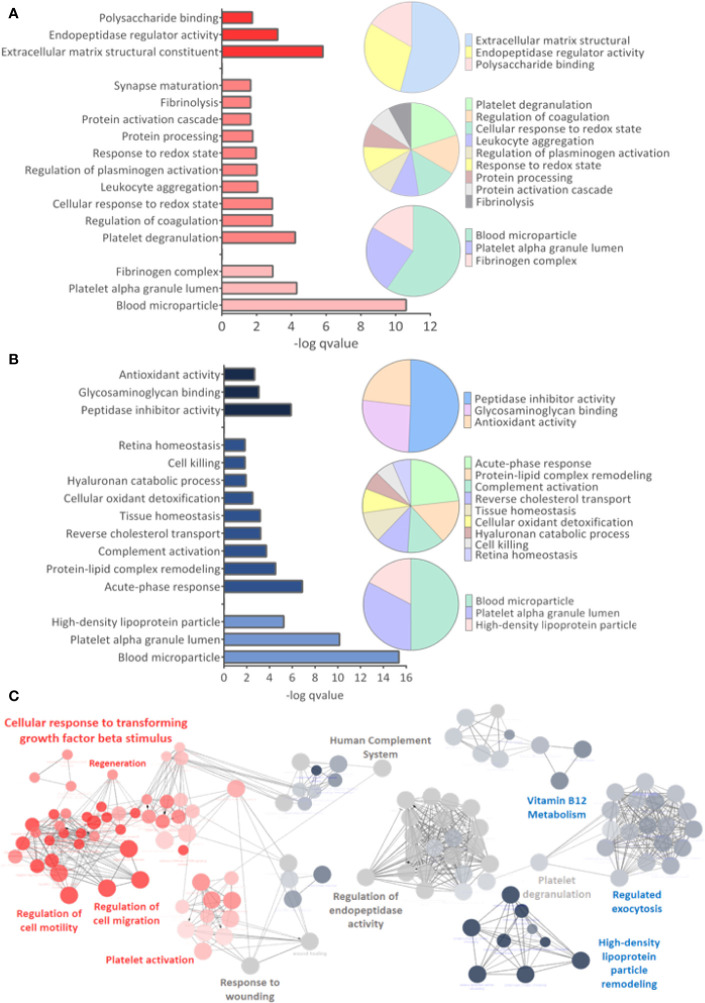
Gene ontology analysis. **(A, B)** From top to bottom are indicated the molecular function (MF), Biological Process (BP), and Cellular Component (CC) related to upregulated and downregulated proteins, respectively. q-value < 0.05. **(C)** Interaction between upregulated (red dots) and downregulated (blue dots) proteins, with their respective related ontologies. The gray color show the ontology related to proteins identified upregulated and downregulated. The analysis was performed in the ClueGo app. Only interactions with a p-value less than or equal to 0.05 were considered, with correction by the Benjamini-Hochberg method. The size of the node shows the significance of the interactions, based on the adjusted p-value.

GO analysis for upregulated and downregulated proteins was performed for molecular function (MF), biological process (BP), and cellular component (CC). Events related to coagulation, leukocyte aggregation, synapse maturation, fibrinolysis, and platelet degranulation are increased in the ZIKV group **(** **Figure 5A** **)**. On the other hand, downregulated proteins are involved with immune responses, tissue homeostasis, and remodeling of the lipid-protein complex. The CC of differently regulated proteins is similar, with emphasis on lipoprotein complexes for downregulated proteins **(** **Figure 5B** **)**. Both upregulated and downregulated proteins showed MFs related to the activities of endopeptidases, indicating higher protease activity in the ZIKV exposed serum. The interaction between proteins upregulated (red dots) and downregulated (blue dots) and the respective ontologies are shown in **Figure 5C**, including TGF-beta signaling and complement activation **(** **Supplementary File 6** **)**.

### Aberrant Pattern of Protease Activity in the Serum of Infants With ZIKV Intrauterine Exposure Compared to Control

**Figure 6:**
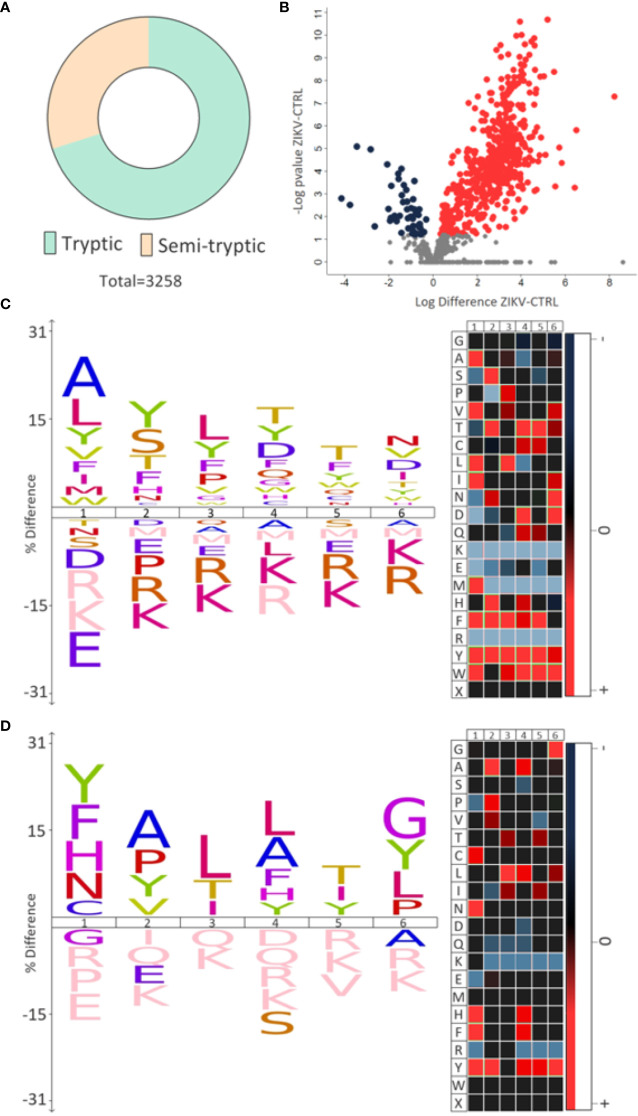
Semi-tryptic peptides. **(A)** The number of tryptic and semi-tryptic peptides identified in samples of depleted serum. q-value < 0.1. **(B)** Volcano plot of differently regulated semi-tryptic peptides. A total of 547 upregulated (red dots) and 47 downregulated (blue dots) were identified. **(C, D)** The most frequent cleavage sites (%) identified by the upregulated and downregulated semi-tryptic peptides, respectively. The numbers 1-6 indicate the position of the amino acids in the peptide sequence and the endopeptidase cleavage site is between positions 1 and 2. In heatmaps, red is the most represented amino acid, while blue is the least represented amino acid.

Semi-tryptic peptides are those cleaved at the C-terminus by trypsin (arginine and lysine residues) and at the other terminal (N-terminus) by another endogenous enzyme. The analysis of semi-tryptic peptides can indicate the action of proteases. In depleted serum, we identified a total of 3,258 peptides and among these, 976 were semi-tryptic **(** **Figure 6A** **)**. To determine the semi-tryptic peptides differentially regulated between the groups evaluated, the same approach described above for proteins was applied. Our results show 547 semi-tryptic peptides upregulated and 47 downregulated in the ZIKV group **(** **Figure 6B** and **Supplementary File 7** **)**; suggesting a higher proteolytic activity in the serum of ZIKV exposed infants. In total, 87 proteases and protease inhibitors were identified in our dataset **(** **Table 2** **)**.

**Table 2:** Proteases and protease inhibitors identified in our dataset.

Gene name	Description
A2M	Alpha-2-macroglobulin
A2ML1	Alpha-2-macroglobulin-like protein 1
ACAA1	3-ketoacyl-CoA thiolase, peroxisomal
ADAMTS13	A disintegrin and metalloproteinase with thrombospondin motifs 13
ALDOA	Fructose-bisphosphate aldolase A
AMBP	Protein AMBP
ANPEP	Aminopeptidase N
AOC2	Retina-specific copper amine oxidase
AOC3	Membrane primary amine oxidase
BCHE	Cholinesterase
BLVRB	Flavin reductase (NADPH)
BTD	Biotinidase
CA1	Carbonic anhydrase 1
CAT	Catalase
CD109	CD109 antigen
CFB	C3/C5 convertase
CFD	C3 convertase activator
CNDP1	Beta-Ala-His dipeptidase
CPB2	Carboxypeptidase B2
CPN1	Carboxypeptidase N catalytic chain
CPN2	Carboxypeptidase N subunit 2
CST3	Cystatin-C
CTBS	Di-N-acetylchitobiase
DBH	Dopamine beta-hydroxylase
DCP1	Dipeptidyl carboxypeptidase I
DNMT3A	DNA (cytosine-5)-methyltransferase 3A
DPEP2	Dipeptidase 2
DPP4	Dipeptidyl peptidase 4
ENPP2	Ectonucleotide pyrophosphatase/phosphodiesterase family member 2
FAP	Prolyl endopeptidase
FKBP1A	Peptidyl-prolyl cis-trans isomerase FKBP1A
FSAP	Factor VII-activating protease
FUCA2	Plasma alpha-L-fucosidase
GGH	Gamma-glutamyl hydrolase
GPLD1	Phosphatidylinositol-glycan-specific phospholipase D
GPX3	Glutathione peroxidase 3
HSPG2	Basement membrane-specific heparan sulfate proteoglycan core protein
ITIH1	Inter-alpha-trypsin inhibitor heavy chain H1
ITIH2	Inter-alpha-trypsin inhibitor heavy chain H2
ITIH3	Inter-alpha-trypsin inhibitor heavy chain H3
ITIH4	Inter-alpha-trypsin inhibitor heavy chain H4
KNG1	Alpha-2-thiol proteinase inhibitor
LCAT	Phosphatidylcholine-sterol acyltransferase
LDHA	L-lactate dehydrogenase A chain
LDHAL6A	L-lactate dehydrogenase A-like 6A
LDHB	L-lactate dehydrogenase B chain
LDHC	L-lactate dehydrogenase C chain
MAN1A1	Mannosyl-oligosaccharide 1,2-alpha-mannosidase IA
MAN2A1	Alpha-mannosidase 2
MASP1	Mannan-binding lectin serine protease 1
MASP2	Mannan-binding lectin serine protease 2
METTL18	Histidine protein methyltransferase 1 homolog
MINPP1	Multiple inositol polyphosphate phosphatase 1
MMP2	72 kDa type IV collagenase
MMP9	Matrix metalloproteinase-9
NAGLU	Alpha-N-acetylglucosaminidase
PCOLCE	Procollagen C-endopeptidase enhancer 1
PCY	Prenylcysteine oxidase 1
PEPD	Xaa-Pro dipeptidase
PGLYRP2	N-acetylmuramoyl-L-alanine amidase
PI16	Peptidase inhibitor 16
PLG	Plasminogen
PON1	Serum paraoxonase/arylesterase 1
PPA2	Inorganic pyrophosphatase 2, mitochondrial
PPIA	Peptidyl-prolyl cis-trans isomerase A
PPIAL4A	Peptidyl-prolyl cis-trans isomerase A-like 4A
PPIAL4C	Peptidyl-prolyl cis-trans isomerase A-like 4C
PPIAL4D	Peptidyl-prolyl cis-trans isomerase A-like 4D
PPIAL4E	Peptidyl-prolyl cis-trans isomerase A-like 4E
PPIAL4F	Peptidyl-prolyl cis-trans isomerase A-like 4F
PPIAL4H	Peptidyl-prolyl cis-trans isomerase A-like 4H
PTGDS	Prostaglandin-H2 D-isomerase
PTPRG	Receptor-type tyrosine-protein phosphatase gamma
PZP	Pregnancy zone protein
QSOX2	Sulfhydryl oxidase 1
RNASE4	Ribonuclease 4
SERPINA1	Alpha-1 protease inhibitor
SERPINA10	Protein Z-dependent protease inhibitor
SERPINA4	Peptidase inhibitor 4
SERPINA5	Plasma serine protease inhibitor
SERPINC1	Antithrombin-III
SERPINF2	Alpha-2-antiplasmin
SERPING1	Plasma protease C1 inhibitor
SOD1	Superoxide dismutase [Cu-Zn]
SOD3	Extracellular superoxide dismutase [Cu-Zn]
TIMP1	Metalloproteinase inhibitor 1
TIMP2	Metalloproteinase inhibitor 2
VNN1	Pantetheinase

Protein description: according to the Swiss-Prot database.

Motif analysis of most frequent cleavage sites for semi-tryptic peptides upregulated in the ZIKV group showed alanine and leucine at the cleaved sites **(** **Figure 6C** **)**. On the other hand, the downregulated semi-tryptic peptides showed tyrosine and phenylalanine as the most frequent cleavage sites **(** **Figure 6D** **)**. The prediction of proteases involved in the cleavage of the all semi-tryptic peptides identified showed that metalloproteinase family is responsible for the cleavage of 24% and plasminogen of 4%. The individual analysis of upregulated and downregulated semi-tryptic peptides showed that metalloproteinases are responsible for the cleavage of 22.8% and 12.6%, respectively **(** **Table 2** and **Supplementary 7** **)**.

**Figure 7:**
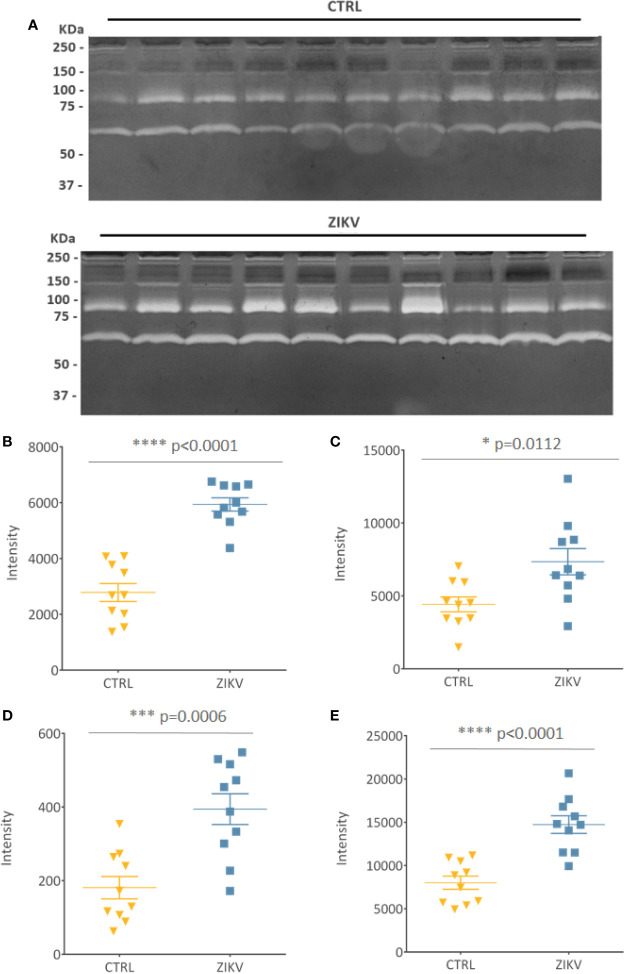
Gelatin zymography. **(A)** Gelatinolytic activity of the samples from the CTRL and ZIKV group, with emphasis on the activities of MMP 9 (92 kDa) and MMP 2 (72 KDa). **(B–D)** The intensities of the molecular weight bands of 72, 92, and 250 kDa, with their respective p-value. **(E)** Shows the sum of all quantified intensities, with their respective p-value.

Evaluation of serum protease activity **(** **Figure 7A** **)** showed an increase in the metalloproteinase 2 (MMP2) **(** **Figure 7B** **)** and metalloproteinase 9 (MMP9) **(** **Figure 7C** **)** in serum from ZIKV group compared to CTRL, which are shown by the intensity of the molecular weight bands of 72 KDa and 92 KDa, respectively **(** **Supplementary File 8** **)**. In addition, the formation of MMPs complexes is indicated by the presence of high molecular weight bands (~ 250 KDa) (Roy et al., 2008) **(** **Figure 7D** **)**. The intensity of all quantified bands was summed, and the result is shown in **(** **Figure 7E** **)**, with p-value <0.0001 **(** **Supplementary File 8** **)**.

## Discussion

### Exposure to ZIKV Is Related to Coagulation, Thrombotic Events, and Late Cerebrovascular Disorders

Quantitative proteomics approaches have been applied to investigate the molecular mechanisms involved in intrauterine ZIKV infection. Infection of primary human neural stem cells (McGrath et al., 2017), neural progenitor cells (Scaturro et al., 2018), and neurospheres derived from induced pluripotent stem cells are used in modeling the effects of first-trimester infections *in vitro* (Garcez et al., 2017; Rosa-Fernandes et al., 2019). In this study, we performed large-scale serum quantitative proteomics to identify molecular changes in infants exposed to ZIKV without early clinical symptoms compared to paired controls, in order to explore and predict molecular evidence of possible late abnormalities during the ongoing follow-up of this cohort.

Proteins related to venous thrombosis and blood coagulation, such as Coagulation factor VII (F7) and Fibrinogen alpha chain (FGA), were upregulated in the ZIKV group. The relationship between viral infections and changes in coagulation processes has already been described for ZIKV (Ramacciotti et al., 2019) and other viruses (Kimmel, 1967; Bibas et al., 2011; da Costa et al., 2012; Goeijenbier et al., 2012; Roy et al., 2013; Wang et al., 2015; Wijarnpreecha et al., 2017; Marques et al., 2017; Neppelenbroek et al., 2018; Ngu et al., 2018; Ramacciotti et al., 2019).

After the occurrence of isolated cases of venous thrombosis in patients who had a positive diagnosis for ZIKV, Ramacciotti et al. (2019) assessed blood D-dimer levels, which are usually monitored for the diagnosis of deep venous thrombosis, of 172 patients who had ZIKV or chikungunya, without cross-infection. The results showed an increase of 19.4% and 63.8% in the D-dimer levels of patients with ZIKV and chikungunya, respectively. Moreover, pro-thrombosis effects related to viral infection of the CNS can result in cerebrovascular complications (Tang et al., 2019). Landais et al. (2017) reported a case of stroke in a 10-month-old child who was positive for ZIKV. A second case of fetal cerebral infarction has been reported, in which a child has been exposed to ZIKV and had no brain changes on MRI. However, at 16 days of age, an area of ​​chronic encephalomalacia was found (Mulkey et al., 2018). A third study confirmed the presence of ZIKV RNA in the placenta, umbilical cord and amniotic membrane on the maternal side of the placenta of a pregnant woman who delivered a child without clinical evidence of microcephaly and CZS. The medical follow-up showed that the child had a stroke on the eighth day of life, even without apparent abnormalities in complete blood count, cranial ultrasound and ammonia values (Raymond and Jakus, 2018). In addition, a report of cerebral vasculitis in an adult patient with ZIKV positive PCR was published (Acevedo et al., 2017). Cerebral vasculitis in neonates is characteristic of congenital diseases (Koeppen et al., 1981; Baskin and Hedlund, 2007; Hauer et al., 2019). Although case reports related to ZIKV exposure are punctual, our study agrees with these findings as we identified proteins related to upregulated cerebrovascular disease in children exposed to ZIKV compared to CTRL group. In our cohort, a patient in the ZIKV group showed changes in MRI and developmental delay. This finding corroborates previous studies applied to the larger cohorts, such as Pomar et al. (2018) and Rice et al. (2018), in which 12.5% and 1.6% of the population exposed to ZIKV who were born without microcephaly and CZS developed complications, respectively. Due do that, we emphasize the importance of medical monitoring of symptomatic and asymptomatic children, as apparently unexplained late neurological complications might be due to exposure to ZIKV in the uterus.

### Cerebrovascular Disorders Related to Increased Activity of Metalloproteinases

MMPs are proteolytic enzymes that degrade the extracellular matrix and basement membranes and participate in biological homeostatic and pathological processes (Birkedal-Hansen et al., 1993). Among the neuronal pathologies linked to MMPs, neuronal apoptosis and oxidative damage to DNA are apparent (Xie et al., 2017). MMPs are expressed in different tissues, including the CNS, where they perform pathological functions linked to the opening of the blood-brain barrier after cerebral ischemia (Cunningham et al., 2005). Reports show that MMPs play a key role in chronic inflammatory diseases of the CNS and participate in the degradation of myelin components (Walker and Rosenberg, 2010). Our study identified an increased activity of MMPs 2 and 9 in the serum of children exposed to ZIKV, as well as an increase in the levels of the Profilin-1 (PFN-1) protein, which is fundamental for myelination (Montani et al., 2014). Neuronal death after cerebrovascular disorders has been reported, and studies show a possible role for MMPs in this phenomenon. Lee (2004) showed increased activity of MMP2 and 9 located mainly in neurons of rats after transient global ischemia. Yang et al. (2010) demonstrated that MMP2 and 9 cleave the proteins Poly(ADP-Ribose) Polymerase 1 (PARP-1) and DNA repair protein XRCC1 (XRCC1), which facilitates oxidative damage in neurons during early ischemia. These proteins are involved in events of cell survival and apoptosis.

### Macular Degeneration and Eye Abnormalities Related Proteins

Ocular complications have already been described in infants diagnosed with CZS (Ventura and Ventura, 2018). Moreover, a recently developed label-free proteomics methodology has also been applied to evaluate the alteration of the ocular protein of children exposed to ZIKV during pregnancy, comparing groups with and without CZS (Rosa-Fernandes et al., 2020). The study showed that the biomolecules involved in neutrophil degranulation, neurodevelopment, cell infiltration, and ocular dysfunction were identified in different abundances between the groups evaluated, being able to discriminate infants exposed to ZIKV during gestation and without early clinical symptoms (Rosa-Fernandes et al., 2020).

In addition, other flaviviruses, such as West Nile fever, transmitted from the mother to the fetus have also been shown to cause eye abnormalities (Alpert et al., 2003). In our analysis, we identified proteins related to premature retinopathy and macular degeneration, such as pigment epithelium-derived factor (SERPINF1), transforming growth factor beta-1 proprotein (TGFB1), fibrinogen alpha chain (FGA), mannan-binding lectin serine protease 1 (MASP1), moesin (MSN), and vitronectin (VTN), upregulated in the ZIKV group, suggesting the possibility of developing these complications. Our analysis of enriched pathways showed that visual phototransduction events are downregulated in this group compared to CTRL. Visual phototransduction is a photochemical and biochemical process that consists of photon absorption by photoreceptor cells, which convert this signal into an electrical cellular response. This electrical response is sent to the brain through action potentials and electrochemical changes. Therefore, the normal activity of this pathway, which is highly conserved in many species, is essential for the proper functioning of vision neurobiology (Mannu, 2014).

Our data together with other reports in the literature, reinforce the possibility of the occurrence of abnormalities related to ZIKV infection, not only including neurological, but also ophthalmological disorders.

The children included in this study are currently monitored by a multidisciplinary clinical team to assess the outcome of exposure to ZIKV. Here, we demonstrate molecular differences between the groups evaluated and we emphasize the activity of MMP2 and MMP9 can emerge as potential biomarkers of exposure to the virus. We have demonstrated the increased activity of MMPs in the cohort exposed to ZIKV, making it possible to apply this finding to monitor MMPs activity in children exposed to ZIKV and who may develop late abnormalities.

## Conclusions

Serum provides information on the entire content of circulating proteins and, as the main fluid available for routine clinical evaluations, has great potential in diagnostic and prognostic analyses. We have shown that children exposed to ZIKV during pregnancy, but who were born without any complications, can present molecular evidence for late abnormalities related to CZS. Our study has shown alterations in proteins that participate in processes related to neuronal death and cerebrovascular abnormalities in the ZIKV group, even if these children do not present clinical evidences of CZS at birth. In addition, vision-related proteins have been identified as downregulated, which may indicate ocular and visual impairments, a frequent characteristic in infants that develop CZS. Another key finding was the increased activity of MMP-2 and MMP-9 in all serum samples in the ZIKV group, which could be associated to neuronal death. The children included in this study are currently monitored by multidisciplinary clinical teams to assess the outcome of long-term exposure to the virus. Our study is the first to assess molecular alterations for late disorders in child victims of the ZIKV epidemic in the Americas, demonstrating that medical follow-up should be carried out on all children exposed to the virus, as late complications can occur.

## Data Availability Statement

The original contributions presented in the study are publicly available. The mass spectrometry proteomics data have been deposited to the ProteomeXchange Consortium via the PRIDE partner repository with the dataset identifier PXD020294.

## Ethics Statement

The studies involving human participants were reviewed and approved by Institutional review board and the ethics committee of the Universidade Federal Fluminense (protocol CAAE number 79890517.6.0000.5243). Written informed consent to participate in this study was provided by the participants’ legal guardian/next of kin.

## Author Contributions

Conceptualization, methodology and project administration : LR-F and GP. Formal analysis: JM-d-S LR-F and GP. Investigation: JM-d-S, LR-F, RB, CA. Resources: RB, FC, RV, PC, ML and CC. Validation: JM-d-S. Visualization: JM-d-S and LR-F. Writing – original draft preparation: JM-d-S, LR-F and GP. Writing – review and editing: all authors. All authors contributed to the article and approved the submitted version.

## Funding

RB was supported by FAPERJ (201.779/2017); GP was supported by FAPESP (2014/06863-3, 2018/18257-1, 2018/15549-1) and CNPq (“Bolsa de Produtividade”). This work was also supported by the VILLUM Center for Bioanalytical Sciences at the University of Southern Denmark.

## Conflict of Interest

The authors declare that the research was conducted in the absence of any commercial or financial relationships that could be construed as a potential conflict of interest.

## Supplementary Material

Supplementary Figure 1 Details of the participants. **(A)** Gestational age (GA) of birth of the study participants. The ZIKV group has nine points, as one participant GA was not noted. **(B)** The age of blood collection for the study. A t-test was applied, and no statistical significance was observed between the groups (p-value = 0.3379). **(C)** Age of the mothers included in the study. **(D)** Circumference of the infants heads in centimeters. The measurements were taken in the first week of the participants life. The ZIKV group has nine points, as one participant head circumference was not noted. **(E, F)** Principal Component Analysis (PCA) for depleted and non-depleted serum, respectively. The yellow and blue dots represent the CTRL and ZIKV groups, respectively. The “Principal Component Analysis” module, available in the Perseus tool, was used to build the graph. Previously, a filter was applied to select only proteins present in all samples.

Supplementary File 1 Demographic, clinical, and radiological characteristics of the study population. Data relating to children included in this study, indicating results of maternal PCR, maternal age, child’s age when collecting serum samples, gestational age of maternal rash, head circumference in the first week of life, gestational age of birth, TORCH analysis, gender, place of residence and clinical follow-up data.

Supplementary File 2 Proteins identified by Proteome Discoverer v2.3.0.523 and PatternLab for proteomics v4.1.1.17 in depleted and non-depleted serum; cellular component analysis, and tissue-related protein. Total signal (NSAF): sum of PatternLab’s normalized label-free quantitation derived from the extracted ion chromatograms (XIC). Adjusted p-value refers to the application of the correction by the Benjamini-Hochberg method. Description: protein description from the Swiss-Prot database. Name: identifier from SwissProt/UniProt database.

Supplementary File 3 Complete information for protein differently regulated between the CTRL and ZIKV groups. Protein ID: Swiss-Prot protein identifier. Fold change: ratio between ZIKV and CTRL protein intensities; negative values indicate greater abundance in the CTRL group compared to the ZIKV group. Protein description: according to the Swiss-Prot database. All proteins satisfy a q-value < 0.1, corrected by Benjamini-Hochberg method. In the analysis of the heat map, the intensities were normalized using the z-score function. Positive values ​​indicate that the data is above average and, when negative, it means that the data is below average.

Supplementary File 4 Disease-related proteins. Complete information from DAVID analysis for all differently regulated, upregulated, and downregulated proteins. GAD_Disease: Gene-Disease Associations.

Supplementary File 5 Full list of pathways disclosed by the GSEA analysis. The results include the pathways identifier, statistical results, and name from Reactome.

Supplementary File 6 Complete information of the DAVID and ClueGO Gene Ontology analysis. CTRL and ZIKV GO information. BP: biological process; CC: cellular component; MF: molecular function.

Supplementary File 7 Analysis of semi-tryptic peptides. Result of the search performed by Proteome Discoverer software. Trypsin was used as a cleavage enzyme (semi-tryptic), allowing a maximum of 2 missed cleavage sites. Description: protein description from the Swiss-Prot database. Name: identifier from SwissProt/UniProt database.

Supplementary File 8 Gelatin zymography. The intensity of the bands corresponds to the results of the ImageLab 3.0 software.
