# Supplementary figures and images for: Serum Proteomics Reveals Alterations in Protease Activity, Axon Guidance, and Visual Phototransduction Pathways in Infants With In Utero Exposure to Zika Virus Without Congenital Zika Syndrome
Source: Front Cell Infect Microbiol. 2020 Nov 18;10:577819. doi: 10.3389/fcimb.2020.577819 (PMC7708324; doi:10.3389/fcimb.2020.577819)

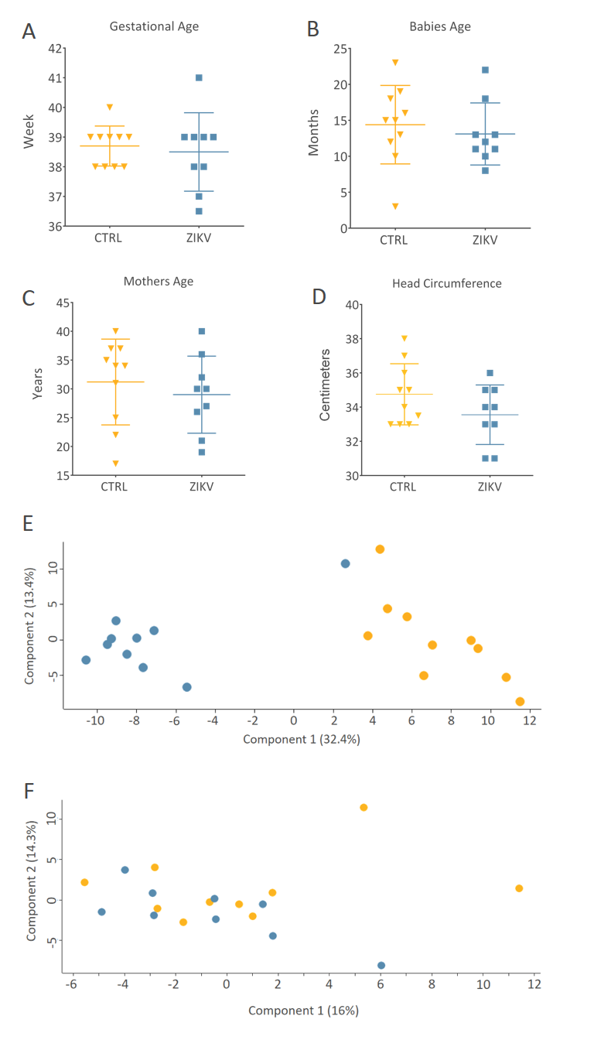

Supplement: Supplementary Figure 1 — Details of the participants. (A) Gestational age (GA) of birth of the study participants. The ZIKV group has nine points, as one participant GA was not noted. (B) The age of blood collection for the study. A t-test was applied, and no statistical significance was observed between the groups (p-value = 0.3379). (C) Age of the mothers included in the study. (D) Circumference of the infants heads in centimeters. The measurements were taken in the first week of the participants life. The ZIKV group has nine points, as one participant head circumference was not noted. (E, F) Principal Component Analysis (PCA) for depleted and non-depleted serum, respectively. The yellow and blue dots represent the CTRL and ZIKV groups, respectively. The “Principal Component Analysis” module, available in the Perseus tool, was used to build the graph. Previously, a filter was applied to select only proteins present in all samples. [file Image_1.tif]
